# Argot2: a large scale function prediction tool relying on semantic similarity of weighted Gene Ontology terms

**DOI:** 10.1186/1471-2105-13-S4-S14

**Published:** 2012-03-28

**Authors:** Marco Falda, Stefano Toppo, Alessandro Pescarolo, Enrico Lavezzo, Barbara Di Camillo, Andrea Facchinetti, Elisa Cilia, Riccardo Velasco, Paolo Fontana

**Affiliations:** 1 Department of Molecular Medicine, University of Padova, via U. Bassi 58/B, 35121, Padova, Italy; 2 Department of Molecular Medicine, University of Padova, via Gabelli 63, 35121, Padova, Italy; 3 Department of Information Engineering, University of Padova, via Gradenigo 6, 35131, Padova, Italy; 4 Istituto Agrario San Michele all'Adige Research and Innovation Centre, Foundation Edmund Mach, via E. Mach 1, 38010, San Michele all'Adige (Trento), Italy

## Abstract

**Background:**

Predicting protein function has become increasingly demanding in the era of next generation sequencing technology. The task to assign a curator-reviewed function to every single sequence is impracticable. Bioinformatics tools, easy to use and able to provide automatic and reliable annotations at a genomic scale, are necessary and urgent. In this scenario, the Gene Ontology has provided the means to standardize the annotation classification with a structured vocabulary which can be easily exploited by computational methods.

**Results:**

Argot2 is a web-based function prediction tool able to annotate nucleic or protein sequences from small datasets up to entire genomes. It accepts as input a list of sequences in FASTA format, which are processed using BLAST and HMMER searches vs UniProKB and Pfam databases respectively; these sequences are then annotated with GO terms retrieved from the UniProtKB-GOA database and the terms are weighted using the e-values from BLAST and HMMER. The weighted GO terms are processed according to both their semantic similarity relations described by the Gene Ontology and their associated score. The algorithm is based on the original idea developed in a previous tool called Argot. The entire engine has been completely rewritten to improve both accuracy and computational efficiency, thus allowing for the annotation of complete genomes.

**Conclusions:**

The revised algorithm has been already employed and successfully tested during in-house genome projects of grape and apple, and has proven to have a high precision and recall in all our benchmark conditions. It has also been successfully compared with Blast2GO, one of the methods most commonly employed for sequence annotation. The server is freely accessible at http://www.medcomp.medicina.unipd.it/Argot2.

## Background

Thanks to the advent of the Next Generation Sequencing technologies, we have assisted to an exponential increase in sequence data generation [1]. The task to assign a curator-reviewed function to every single sequence is unworkable, calling for efficient/effective methods to assign automatic annotation are necessary as a first analysis step to support working hypotheses and drive experimental validations of biological functions.

Computational approaches can be rather imprecise because functional inference is not as straightforward as one would expect, due to the unevenness of the classical paradigm "sequence-structure-function". Some authors suggest that for sequences sharing less than 30% of identity, the functional transfer may be highly inaccurate or completely wrong [2,3]: in particular Enzyme Classification (EC) numbers tend to be conserved only for proteins with sequence identity above 80%. Other authors report different figures [4,5] confirming the difficulty to agree on a unique view due to a certain unpredictability of biological systems.

In the category of sequence-based methods, the simple search for homologous sequences is considered a common practice for function prediction based on annotation transfer, and BLAST [6] can be considered a gold standard. If its classical pairwise alignment engine fails, the profile based PSI-BLAST [6] is able to identify relationship among distantly related proteins.

Another widely accepted approach relies on functional domains assignments. HMMER [7], which is based on Hidden Markov Models (HMM), is among the most known tools falling in this category. HMMER is mainly used to query the Pfam HMM models [8] and search for functional patterns and domains in the target sequences.

Recently, the Gene Ontology (GO) consortium [9] has revolutionized the way to access knowledge data and has rapidly become a standard *de facto*. The GO is organized in a hierarchical directed acyclic graph that greatly facilitates the mining of biological information by computational algorithms.

With the advent of GO and UniProtKB-GOA database (GOA) [10] of functionally annotated proteins, several algorithms have been developed to improve functional inference based on the plain use of BLAST [11]. Among these solutions, Blast2GO [12-14] can be considered one of the best platforms to assist the user in annotating sequences.

In this paper, we present Argot2 (Annotation Retrieval of Gene Ontology Terms), a tool designed for high-throughput annotation of large sequence data sets with high efficiency and precision. Argot2 is born for in-house needs to annotate predicted genes from large-scale sequencing projects; now it has a free and fully functional web interface and its engine has been completely revisited. It has been extensively tested during highly challenging endeavours as grape [15] and apple [16] genome annotations and it has been continuously refined from its early version, Argot [17], to reach a high flexibility and confidence in extracting fruitful knowledge from different sources of information. The web server version is computationally efficient, highly scalable, and it is able to address the different needs of basic and advanced users in annotating small sets of proteins up to entire genomes. Here we also report the assessment of Argot2 tested in four different configurations and in comparison with Blast2GO.

## Methods

### Algorithm description

Argot2 processes the GO annotations of the hits retrieved by BLAST and HMMER searches. A weighting scheme and a clustering approach are applied to select the most accurate GO terms for annotating the target proteins.

Argot2 takes a list of GO terms belonging to the GO graph *G(V,E) *as input and weights them according to the e-value score of the hits. Assuming that the set *V *is ordered, it is possible to establish a one-to-one correspondence between the *i^th ^*GO term *g_i _*∈ *V *used for the annotation, its weight *w_i _*and the e-value scores *S_i _*and *S_i_' *given by BLAST and HMMER. The weights are computed as follows:

(1) $$w_{i}=-\log\left(S_{i}\right)\;\text{for}\;\text{BLAST}$$

(2) $$w_{i}=-\log\left(S_{i}^{\prime }\right)\cdot f\left(\frac{1}{P}n_{g_{i}}\right)\;\text{for}\;\text{HMMER}$$

As pfam2go [18] provides a minimal coverage of GO terms for each Pfam model, we extract from GOA the GO annotations of all proteins belonging to each Pfam entry to enrich these assignments. In Eq. 2 $\frac{1}{P}n_{g_{i}}$ is the frequency of the GO term *g_i _*calculated over the total number *P *of proteins in the model and *f*(*x*) is a logistic curve introduced to reward highly frequent terms and to penalize those that are sparse and likely false positives.

**Figure 1:**
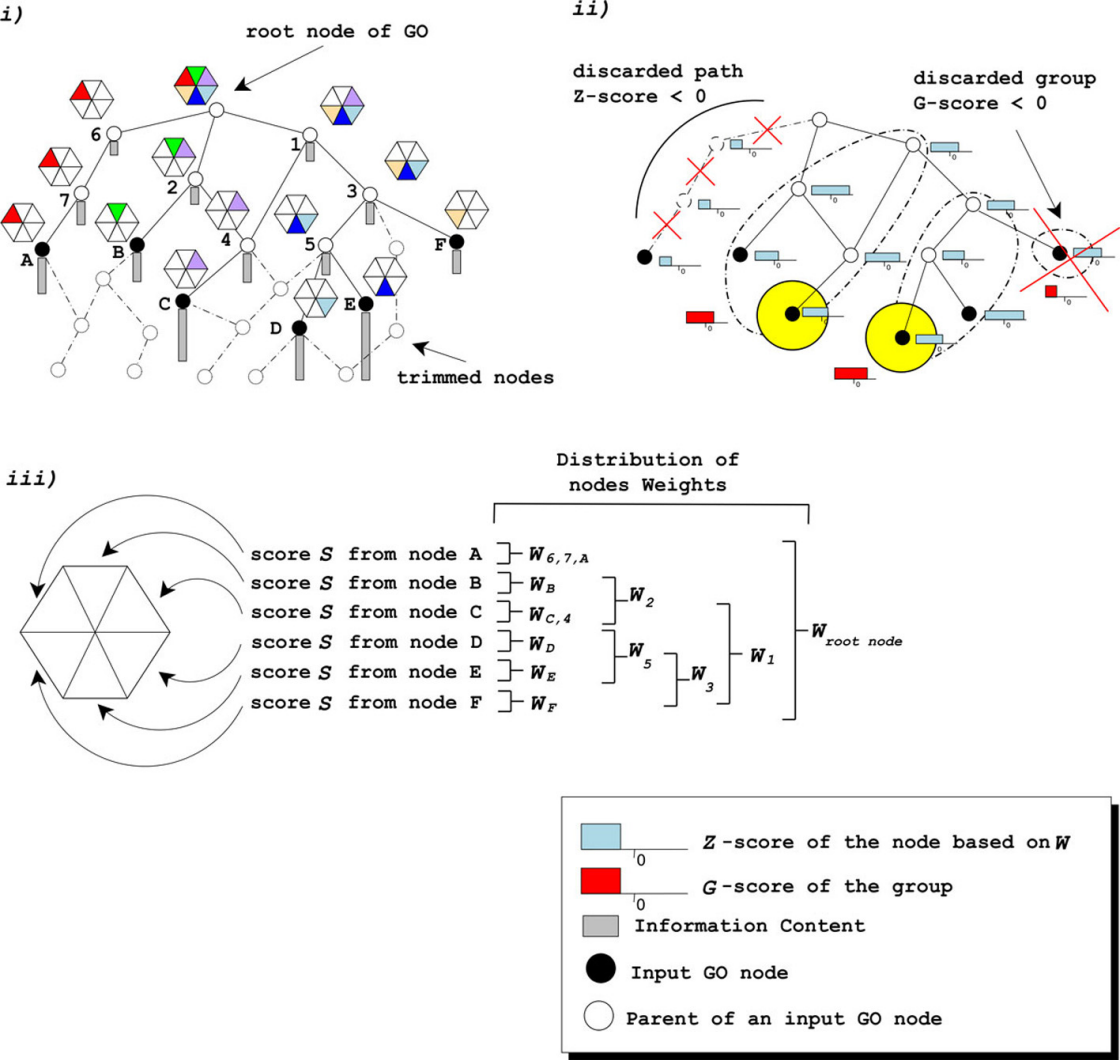
**Argot2 algorithm**. i) Position of the retrieved nodes in the GO graph (black circles) with their weights (W). White circles connected by dashed lines are pruned GO terms that are not present in the final GO-slim. ii) Filtering steps based on Z-score and G-score (see the main text). The yellow big circles are the representatives of the corresponding groups having the highest Total Score (TS) and are used for the annotation. iii) The hexagons report the cumulative weights of the GO nodes i.e. W_2 _is obtained by the sum of its child nodes marked as black circles (W_B _and W_C_). Node 4 does not contribute to the cumulative score, as it is a reconstructed parent from node C. It inherits the weight of node C only (W_C,4_).

All the possible paths starting from the input GO terms and leading to the root node are reconstructed and the GO nodes not included in any of these paths are discarded from the analysis, obtaining the so-called "GO-slim" (Figure 1-i).

The remaining GO terms are grouped together in sets *Gr_k _*∈ ℘;(*V*) according to their semantic similarity [19]: the nodes that share a strong biological relationship form a unique informative group, and only the most specific and high scoring annotations are considered.

Given two generic GO terms *g_i_*, *g_j _*∈ *V*, we use the Lin's formula [20] (Eq. 3) as a semantic similarity measure. This metric has been chosen since it gave the best results in clustering annotations with respect to other existing methods [17].

The Lin's formula is defined as:

(3) $$sim\left(g_{i},g_{j}\right)=\frac{2\cdot sim_{res}\left(g_{i},g_{j}\right)}{IC\left(g_{i}\right)+IC\left(g_{j}\right)}$$

In this formula, the function *sim_res _*: *V *× *V *→ ℜ defined as: $sim_{res}\left(g_{i},g_{j}\right)=max_{g\in S\left(g_{i},g_{j}\right)}\left\{IC\left(g\right)\right\}$ represents the highest Information Content *IC *among the subsumers of the terms *g_i _*and *g_j_*. Using the notation *g_i _*↦ *g_j _*to mean that a path from the term *g_i _*to the term *g_j _*exists, the set of the subsumers can be defined through the function *S*: *V *× *V *→ ℘(*V*) as *S*(*g_i_*,*g_j_*) = {*g *∈ *V*:*g *↦ *g_i _*^ *g *↦ *g_j_*}.

The function *IC *: *V *→ ℜ is the Information Content of the *i^th ^*GO term calculated according to the Resnik formula [21] as:

$$IC\left(g_{i}\right)=-\log\left(\frac{\left|\left\{g:g_{i}\mapsto g\right\}\right|}{\left|\left\{g:g\in GOA\right\}\right|}\right)$$

where |{*g*:*g_i _*↦ *g*}| indicates the total number of occurrences of GO terms descending from GO term *i *and |{*g*: *g *∈ *GOA*}| is the total number of GO terms in the GOA database.

Three scores are then introduced to filter isolated GO terms and to rank the remaining ones. The first one, the Group Score *GrS *: *N *→ ℜ, is the sum of the cumulative Internal Confidence *InC *of the nodes *g_j _*belonging to the *k^th ^*group *Gr_k_*, being *N *the set of the natural numbers:

$$\text{GrS(k)}=\underset{\left\{j:\;g_{j}\in Gr_{k}\right\}}{\sum }InC\left(g_{j}\right)$$

The Internal Confidence *InC *: *V *→ ℜ is a cumulative measure that takes into account the global cumulative weight distributions *W*: *V *→ ℜ defined as $W\left(g\right)=\underset{\left\{j:\;g\mapsto g_{j}\right\}}{\sum }w_{j}$, that is the sum of the weight *w *of a GO term *g *(Eq. 1 and Eq. 2) plus the weights of its children, and the sum of the cumulative weight of the root node (see Figure 1-iii):

(4) $$InC\left(g_{i}\right)=\frac{\underset{\left\{j:\;g_{i}\mapsto g_{j}\right\}}{\sum }w_{j}}{\underset{\left\{j:\;g_{root}\mapsto g_{j}\right\}}{\sum }w_{j}}=\frac{W\left(g_{i}\right)}{W\left(g_{root}\right)}$$

The second score *Z*: *V *→ ℜ, called Z-score, is calculated for each extracted GO term *g_i _*as follows:

$$Z\left(g_{i}\right)=\frac{W\left(g_{i}\right)-\bar{W}}{\sigma }$$

where $\bar{W}$is the weight of the root node divided by the total number of the retrieved GO nodes, while *σ *is the standard deviation of all the weights.

If the Z-score and the Group Score are below a certain threshold, the corresponding GO terms are discarded. These filtering steps reward those paths, up to the root, that are statistically significant discarding the branches of the GO graph containing nodes with low weights (see in Figure 1-ii the discarded path and group).

After the filtering phases, the algorithm assigns the third score, the Total Score *TS *: *V *→ ℜ, to each culled GO term *g_i_*, according to the following formula:

(5) $$TS\left(g_{i}\right)=IC\left(g_{i}\right)\cdot InC^{nc}\left(g_{i}\right)\cdot \frac{InC^{nc}\left(g_{i}\right)}{GrS^{nc}\left(g_{i}\right)}\cdot w_{i}$$

where *Inc^nc^*: *V *→ ℜ is the non-cumulative internal confidence, calculated as

(6) $$InC^{nc}\left(g_{i}\right)=\frac{w_{i}}{\underset{\left\{j:\;g_{root}\mapsto g_{j}\right\}}{\sum }w_{j}}=\frac{w_{i}}{W\left(g_{root}\right)}$$

Differently from the cumulative Internal Confidence *InC *defined by (Eq. 4), it estimates the local non-cumulative weight distribution, which considers only the weight of the term under analysis.

The function *GrS^nc ^*: *N *→ ℜ is the non-cumulative Group Score associated to the *k^th ^*group *Gr_k_*. It is calculated as the sum of the non cumulative Internal Confidence *InC^nc ^*(Eq. 6) of the nodes belonging to that group:

$$GrS^{nc}\left(k\right)=\underset{\left\{g_{j}\in Gr_{k}\right\}}{\sum }InC^{nc}\left(g_{j}\right)$$

The GO terms with *TS *above a chosen threshold are extracted and reported.

The score rewards those hits that are particularly significant and specific, thanks to the contribution of the Information Content (see yellow circles in Figure 1-ii).

The non cumulative measures *InC^nc ^*and *GrS^nc ^*have been introduced to guarantee that no biases are introduced due to the scores of child nodes.

### Web server functionalities and features

Argot2 has been completely reengineered to speed up and improve the annotation process. The algorithm has undergone several adjustments to easily merge the GO annotations retrieved from different databases. UniProt [22] and Pfam are presently used as reference databases and queried using BLAST and HMMER respectively.

**Figure 2:**
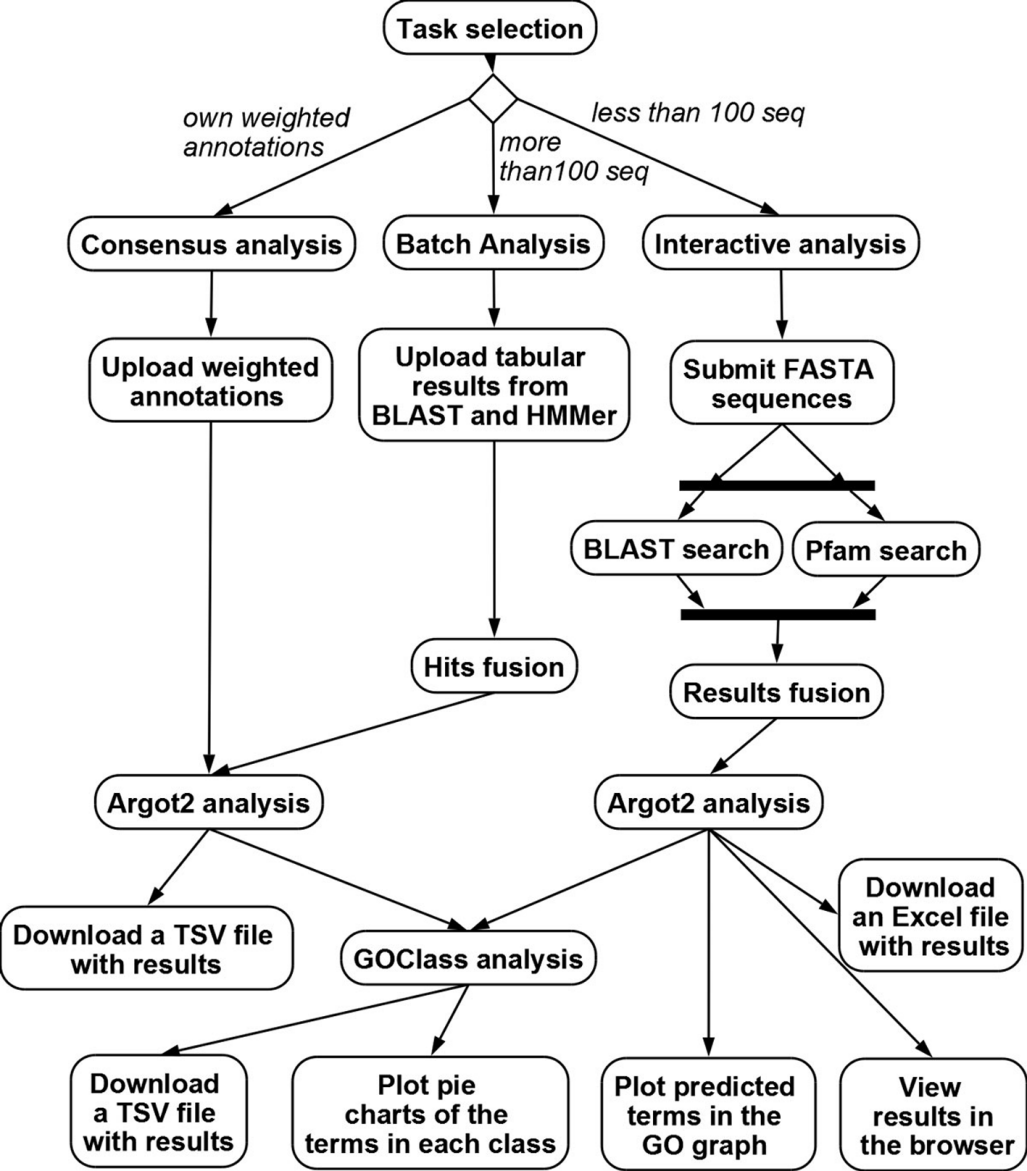
**Activity diagram of the Argot2 web server**. Activity diagram of the Argot2 web server showing the three types of access: "Interactive analysis" for up to 100 sequences, "Batch analysis" for more than 100 sequences and "Consensus analysis" based on provided weighted GO annotations (see the main text).

The server can be accessed in three ways addressing different needs from small to large scale function predictions (see Figure 2).

a) In the "interactive analysis" the user simply inputs up to 100 DNA/protein sequences in FASTA format. For every sequence, a table is shown containing: predicted annotations with scores, hyperlinks to external sources, lists of proteins contributing to the final annotation, and a graphical position map of the retrieved hits into the GO graph.

b) The "batch analysis" is addressed to researchers interested in the annotation of entire genomes. Since this process is highly demanding, due to the long computational time required by BLAST and HMMER, we ask users to perform BLAST and HMMER searches locally and then upload search results into Argot2.

c) The last access option is called "consensus analysis" as users may provide their own weighted GO terms for each protein; these annotations can be obtained by any other method or database, in addition or in alternative to the "default" BLAST and HMMER searches used by the web server. The outputs of the analyses of type *b *and *c *are Excel or Tab Separated Values (TSV) files listing the retrieved annotations along with specific metrics: Total Score, Information Content and Internal Confidence. Finally, predictions can be automatically clustered in functional classes by using the GOClass tool (Additional file 1) and viewed as pie-charts. The Argot2 algorithm steps are mainly based on the original idea published in [17]. Important changes have been applied to the procedure to filter potential false positive hits out during the evaluation of the predicted terms. The raw measure Total Score (TS) has also been redefined. The server is freely accessible at the URL in [23].

### Argot2 assessment

Argot2 has been benchmarked in four different conditions to test how proteins (either kept or removed from the databank) influence the results, and which is the impact of domain based HMM searches. The four different configurations are indicated in the following as: a) BH_with, b) B_with, c) BH_without, d) B_without. The prefix "BH" means that Argot2 has been tested on BLAST and HMMER searches, whereas "B" only on BLAST searches. The suffix "with" means that the proteins of the test set are present in the databank and "without" means they have been eliminated. Argot2 has been also compared with Blast2GO.

The assessment of Argot2 was based on the guidelines of the "Critical Assessment of Function Annotations" (CAFA) experiment [24] (see Additional file 7). We tested over 4000 proteins with already available GO annotations in GOA, both from Eukaryota (Euk) and Prokaryota (Pro), randomly extracted from about 50000 sequences released for the CAFA challenge. In addition, the well annotated yeast genome, comprising 6187 annotated proteins, has also been used as a test set. The details and statistics of the test sets are available in Additional files 2, 3, 4, 5 and on our web site [25].

The evaluation has been carried out at a protein-centric level using the following criteria. Let *N *be a pool of unknown target proteins. For each given protein *p*, the GO terms predicted by each method are retrieved and ranked accordingly to the corresponding Total Score *TS_p _*(Eq. 5).

For a given threshold *t *applied to the Total Score *TS_p _*the four different configurations are assessed based on precision and recall, calculated for each protein *p *as:

(7) $$PR_{p}^{t}=\frac{TP_{p}^{t}}{TP_{p}^{t}+FP_{p}^{t}}\;;\;RC_{p}^{t}=\frac{TP_{p}^{t}}{TP_{p}^{t}+FN_{p}^{t}}$$

The number of True Positives ($TP_{p}^{t}$) is the size of the intersection between the sets of benchmark (true) and predicted GO terms with score *TS_p _> t*. The number of False Positives ($FP_{p}^{t}$) is the size of the difference between the sets of predicted and true GO terms. The number of False Negatives ($FN_{p}^{t}$) is the size of the difference between the sets of true and predicted GO terms. The denominators of (Eq. 7) and (Eq. 8) represent the total number of predicted terms and the number of true terms, respectively. If, for a given threshold *t*, a protein has not any annotated term, its precision is not calculated.

### Assessment Method 1 (m1) with sliding threshold

We consider a set of threshold scores *t *ranging from 0 to the maximum observed score *t_max_*. For each *t*, precision and recall are averaged across the *N *proteins of the pool, obtaining:

$$PR^{t}=\frac{1}{N}\sum_{p=1}^{N}PR_{p}^{t}\;;\;RC^{t}=\frac{1}{N}\sum_{p=1}^{N}RC_{p}^{t}$$

Each pair of values (1-*PR^t^, RC^t^*) represents a point of the precision/recall curve.

### Assessment Method 2 (m2) with sliding threshold

We calculate precision and recall as in the case of m1 method, but all the GO terms retrieved by the different tools (predicted terms) and those originally annotated on the benchmark proteins (true terms) are first propagated to the root. Thus, all GO terms standing in the paths of the predicted/true terms up to the root are considered in the assessment. The idea is that a predicted GO term, though not exact, may share some of its parent nodes with some parent nodes of one true GO term. This term cannot be considered completely wrong but rather closely related and, consequently, its shared parent nodes are included in the evaluation. See Additional file 7 for details and extensive explanation of assessment m1 and m2.

## Results and discussion

**Figure 3:**
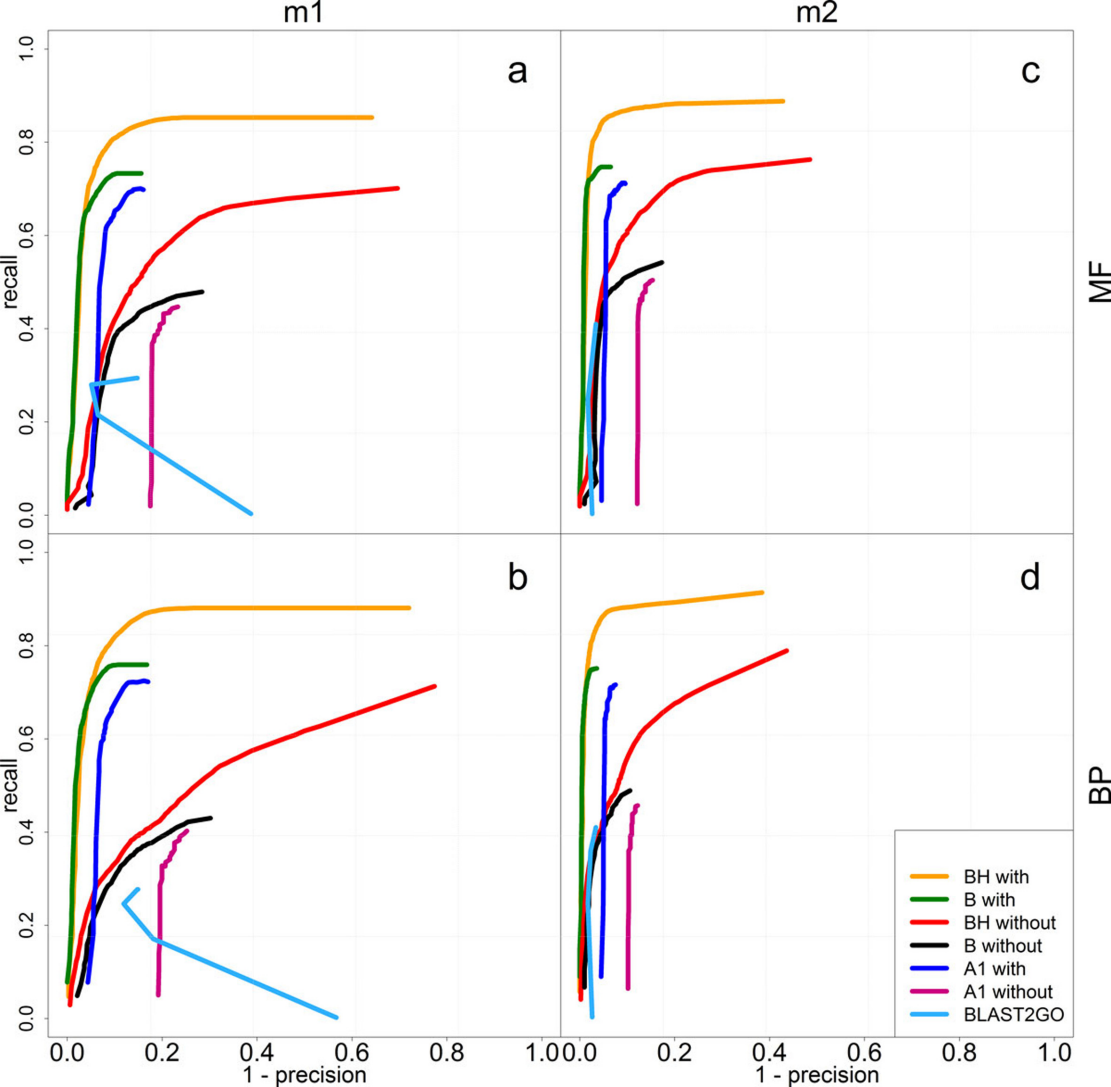
**Precision/recall curves of the yeast benchmark test**. Precision/recall curves for Molecular Function (MF) and Biological Process (BP) calculated with method m1 and m2 (see the main text) for yeast test set. Recall and 1-precision of the tested algorithms are reported in y-axis and x-axis, respectively, for the two configurations "with" (keeping the benchmarked proteins in the databank) and "without" (removing them from the databank). See the main text for the abbreviations BH_with, B_with, BH_without, and B_without.

Precision/recall curves for Molecular Function (MF) and Biological Process (BP) have been calculated with method m1 and m2 for yeast (Figure 3), Eukaryota, and Prokaryota test sets (Additional file 3 and 4). The first outcome of our benchmarking provides evidence of the effectiveness of the combination of BLAST and HMMER weighted hits (BH vs. B curves shown in Figure 3) in recovering a large number of GO terms (high recall), without significantly affecting precision, and outperforming the use of BLAST alone.

One potential bias in the assessment of all methods is that 84% and 99% of the proteins, in Euk and Pro test sets respectively (Additional file 2), are annotated without manual validation (Inferred from Electronic Annotation, IEA) and an over-estimation of tool performance may occur due to the use of predicted terms for functional inference [26]. To investigate the influence of this potential bias, the yeast proteome was used as benchmark, since a wealth of experimental data is available for this organism (over 84% of the proteins contain at least one non-IEA annotation. See Additional file 2). Though this is a challenging task involving 6187 sequences, the assessment gives an idea of what Argot2 is expected to do on a genome scale, namely to obtain a precise and thorough picture of molecular functions and biological processes of an entire organism. The general trends and the robustness shown in Pro and Euk test sets are confirmed (see Additional file 3, 4, and 6 from "a" to "h"). Nonetheless, a minor decrease in performance can be observed in yeast. This is due to the fact that yeast is mainly annotated with highly informative non-IEA GO terms, whose frequency in GOA databank is very low and consequently their retrieval may be a hard task. In particular it is possible to observe that the recall worsen, whereas the precision is only marginally affected proving that Argot2 is able to retrieve reliable and even low-frequent GO terms (compare for example the third column of Additional file 6 with the first two columns of the same figure, row by row).

This trend is confirmed when target proteins are removed from the databanks used to train Argot2 (see curves suffixed by "with" vs. those suffixed by "without" in Figure 3). This issue is not present in Pro and Euk test sets, which mainly include highly frequent IEA GO terms. As expected, results of Pro and Euk test sets get slightly worse (see Additional file 3 and 4), but yeast is more affected and Argot2 finds more difficulties in extracting the right GO terms (see Figure 3, column "m1"). Nevertheless, method m2 reveals that, in these critical situations, Argot2 tends to be conservative rather than inaccurate, i.e. to show a lower recall but still a good precision (see Figure 3: "a" vs. "c", "b" vs. "d", "BH_without" and "B_without"). In conclusion, the lower performance is due to shallowness rather than inaccuracy. This means that most of the predicted nodes, though approximate, fall into the path of the correct annotations.

Finally, some interesting conclusions can be drawn in the "one-to-one" comparison with Blast2GO using the B_with Argot2 version that exploits the same BLAST data of Blast2GO. According to benchmark "m2", the recall is generally higher for Argot2 whereas the precision is comparable, to some extent, between the two tools. However, Argot2 is more effective in retrieving the exact original annotations, as evidenced by the use of assessment method m1 (see "m1" column in Figure 3 and "m1" rows in Additional file 6). The irregular contour trend of Blast2GO may be due to the sliding "Annotation Cut-off" parameter, which does not seem to be a well discriminating score. Fine tuning may be required, even though the default value suggested for the parameter "Annotation Cut-off", i.e. 55, gives the best trade-off between precision and recall. Moreover, Argot2 is fairly more computationally efficient compared to Blast2GO. Starting from BLAST and HMMER results, which remain the limiting steps of the process, Argot2 takes only few hours to annotate an entire genome.

## Conclusions

Argot has been revisited to increase both accuracy and precision, thanks to an improved weighting scheme and the introduction of Pfam models. The server automatically downloads new releases of the used databanks UniProtKB-GOA, UniProt, Gene Ontology, and Pfam on a monthly basis to give end users an updated access to the tool. Presently, in our testing conditions Argot2 performs reasonably well in terms of both precision and recall, showing that TS score can effectively discriminate among false and true positives. The main rationale has been to create a tool able to favour the precision with respect to the recall. This is critical when annotating very large genome data sets, since reducing the false positives rate is definitely desirable. This can prevent biased information from impacting negatively on post-genome studies and statistics. In addition, we plan to associate a p-value to the raw score TS and to add new sources of information, trying to give an answer to non-trivial cases that lie in the twilight zone beyond similarity based evidences. In future releases we could explore other metrics, for example to assess different semantic similarity measures and to compare their performances with Lin's formula currently used by Argot2 (see [27]).

## List of abbreviations used

*All abbreviations in the text excluded from the following list are specific of this paper and have been defined in the main text*.

BLAST: Basic Local Alignment Search Tool; GO: Gene Ontology; GOA: Gene Ontology Annotation; EC: Enzyme Classification; PSI-BLAST: Position-Specific Iterative Basic Local Alignment Search Tool; HMM: Hidden Markov Model; TSV: Tab Separated Values; URL: Uniform Resource Locator; CAFA: Critical Assessment of Function Annotations; TP: True Positive; FP: False Positive; FN: False Negative; MF: Molecular Function; BP: Biological Process; IEA: Inferred by Electronic Annotation.

## Competing interests

The authors declare that they have no competing interests.

## Authors' contributions

Critical revision of the manuscript for important intellectual input: ST, RV, PF, BDC. Technical and material support: MF, EC, EL, AF. Study supervision: PF and ST. Study concept: ST, MF, BDC, PF. Architectural design: MF, ST. Software development: MF, PF, AP and EC. Drafting of the manuscript: ST, BDC and PF. All authors read and approved the final manuscript.

## Supplementary Material

Additional file 1 **GOClass algorithm details**. Details of the GOClass algorithm used to cluster the GO terms, and more general views of the results obtained by Argot2. Click here for file

Additional file 2 **Datasets statistics**. Parameters used in the benchmarks and some statistics about the datasets. Click here for file

Additional file 3 **Precision/Recall curves for the Eukaryota dataset**. Precision/recall curves for Molecular Function (MF), Biological Process (BP) and Cellular Component (CC) calculated with methods m1 and m2 for Eukaryota test set. Click here for file

Additional file 4 **Precision/Recall curves for the Prokaryota dataset**. Precision/recall curves for Molecular Function (MF), Biological Process (BP) and Cellular Component (CC) calculated with methods m1 and m2 for Prokaryota test set. Click here for file

Additional file 5 **Precision/Recall curves for the Yeast dataset**. Precision/recall curves for Molecular Function (MF), Biological Process (BP) and Cellular Component (CC) calculated with methods m1 and m2 for Yeast test set. Click here for file

Additional file 6 **Precision/Recall curves for the Euk, Pro and Yeast datasets**. Precision/recall curves for Molecular Function (MF) and Biological Process (BP) calculated with methods m1 and m2 for Euk, Pro and Yeast test sets. Click here for file

Additional file 7 **CAFA guidelines explanation**. Document that explains m1 and m2 methods using a simple example. Click here for file
