# Supplementary material for: Argot2: a large scale function prediction tool relying on semantic similarity of weighted Gene Ontology terms
Source: BMC Bioinformatics. 2012 Mar 28;13(Suppl 4):S14. doi: 10.1186/1471-2105-13-S4-S14 (PMC3314586; doi:10.1186/1471-2105-13-S4-S14)
Supplement: Additional file 1 — GOClass algorithm details. Details of the GOClass algorithm used to cluster the GO terms, and more general views of the results obtained by Argot2. [file 1471-2105-13-S4-S14-S1.pdf]

GOClass algorithm details

In order to obtain more general views of the annotations found, a clustering algorithm named GOClass has been designed. The aim is to find generic functional classes that can group proteins according to their shared annotations. Each GO term belonging to the annotated protein is traced back up to the root node. During the path reconstruction, a parent node will be considered as the generic "class node" (see azure circle in Fig 1-ii) for that GO term if it has an *IC* lower than the chosen cut-off. In this way, a sort of GO terms grouping/clustering occurs. As shown in Fig. 1 "class nodes" 1, 2, 3, and 4 subsume and consequently cluster the GO terms belonging to proteins *A*, *B*+*C*, *D*+*C*, and *E* respectively. Due to the distribution of annotations and graph connections, GO terms belonging to the same protein can be part of different classes as shown for protein *C* whose nodes belong to both class 2 and 3 (see Fig. 1). In this case, the protein is inevitably numbered both in class 2 and class 3.
In the path reconstruction the user can select only those GO predictions having at least a certain TS score (the default is set to 2.0) and can choose a certain *IC* cut-off. The higher the *IC* cut-off (up to 1.0) the more classes will be returned whereas the lower the *IC* cut-off (up to 0.0 corresponding to the root node and consequently the grouping into the "root" class) the more general classes will be retrieved. We suggest an *IC* of 0.1 as default.

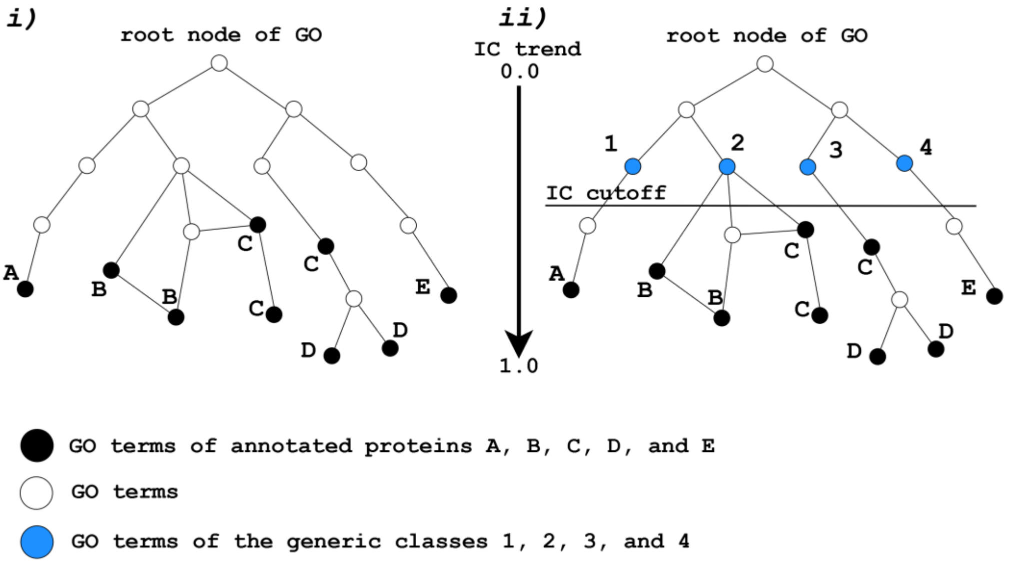

Figure 1: GOClass algorithm example run.
