# Supplementary material for: Argot2: a large scale function prediction tool relying on semantic similarity of weighted Gene Ontology terms
Source: BMC Bioinformatics. 2012 Mar 28;13(Suppl 4):S14. doi: 10.1186/1471-2105-13-S4-S14 (PMC3314586; doi:10.1186/1471-2105-13-S4-S14)
Supplement: Additional file 2 — Datasets statistics. Parameters used in the benchmarks and some statistics about the datasets. [file 1471-2105-13-S4-S14-S2.pdf]

Benchmark details

Databanks Releases

The table below reports the database releases used for benchmarking.

DATABANK	RELEASE DATE
**GOA**	13.12.2010
**GeneOntology OBO file**	24.12.2010
**Pfam (Pfam-A + Pfam-B)**	07.10.2009
**UniProt (Trembl + SwissProt)**	30.11.2010

Parameters of the algorithms

Argot (http://mbi.bio.unipd.it/argot_download) and Blast2GO version 2.4.6 have been used with default parameters.
The Total Score (TS) of Argot 1.1 is different from TS of Argot2. The parameter name has been kept only for historical reasons (see Argot (Fontana, et al., 2009) and "Algorithm details" above).
The sliding threshold used for generating the precision/recall plots are:
Argot: TS ranging from 0 up to 30
Argot2: TS ranging from 0 up to 20000
Blast2GO: "Annotation CutOff" ranging from 0 up to 90
The suggested defaults for these parameters are:
Argot: TS = 3
Argot2: TS = 200
Blast2GO: "Annotation CutOff" = 55

Benchmark test sets characteristics

The table below reports the distribution of the "evidence codes" associated to the GO terms used for the annotation of the Yeast, Pro and Euk test sets. To notice the occurrence of "non-IEA" (i.e. GO terms that are associated with any evidence code but IEA) that are rare in GOA databank with respect to "IEA" (i.e. GO terms that are associated with IEA evidence code).

	**Yeast (*S. cerevisiae*) statistics**	**Pro (Prokaryota) statistics**	**Euk (Eukaryota) statistics**
**Evidence codes**	**Occurrence**	**Occurrence**	**Occurrence**
**EXP**	26	-	7
**IEA**	35372	12431	11259
**IEP**	134	-	23
**IGI**	2515	-	8
**NAS**	149	-	117
**IPI**	4981	6	22
**ND**	6	-	97
**IC**	398	-	2

**RCA**	-	-	2
**ISS**	69	10	178
**IMP**	6560	10	30
**IDA**	13075	18	226
**TAS**	2008	-	122
**Total GO terms In the Test Set**	65293	12475	12093
**Total proteins in the Test Set**	6187	2000	2000
**Fraction of proteins in the Test Set containing at least one NO-IEA term or only IEA terms**			
**NO-IEA**	5204	21	314
**NO-IEA (%)**	84%	1%	16%
**IEA**	983	1976	1686
**IEA (%)**	16%	99%	84%
