# Supplementary figures and images for: Argot2: a large scale function prediction tool relying on semantic similarity of weighted Gene Ontology terms
Source: BMC Bioinformatics. 2012 Mar 28;13(Suppl 4):S14. doi: 10.1186/1471-2105-13-S4-S14 (PMC3314586; doi:10.1186/1471-2105-13-S4-S14)

Euk

Pro

Yeast

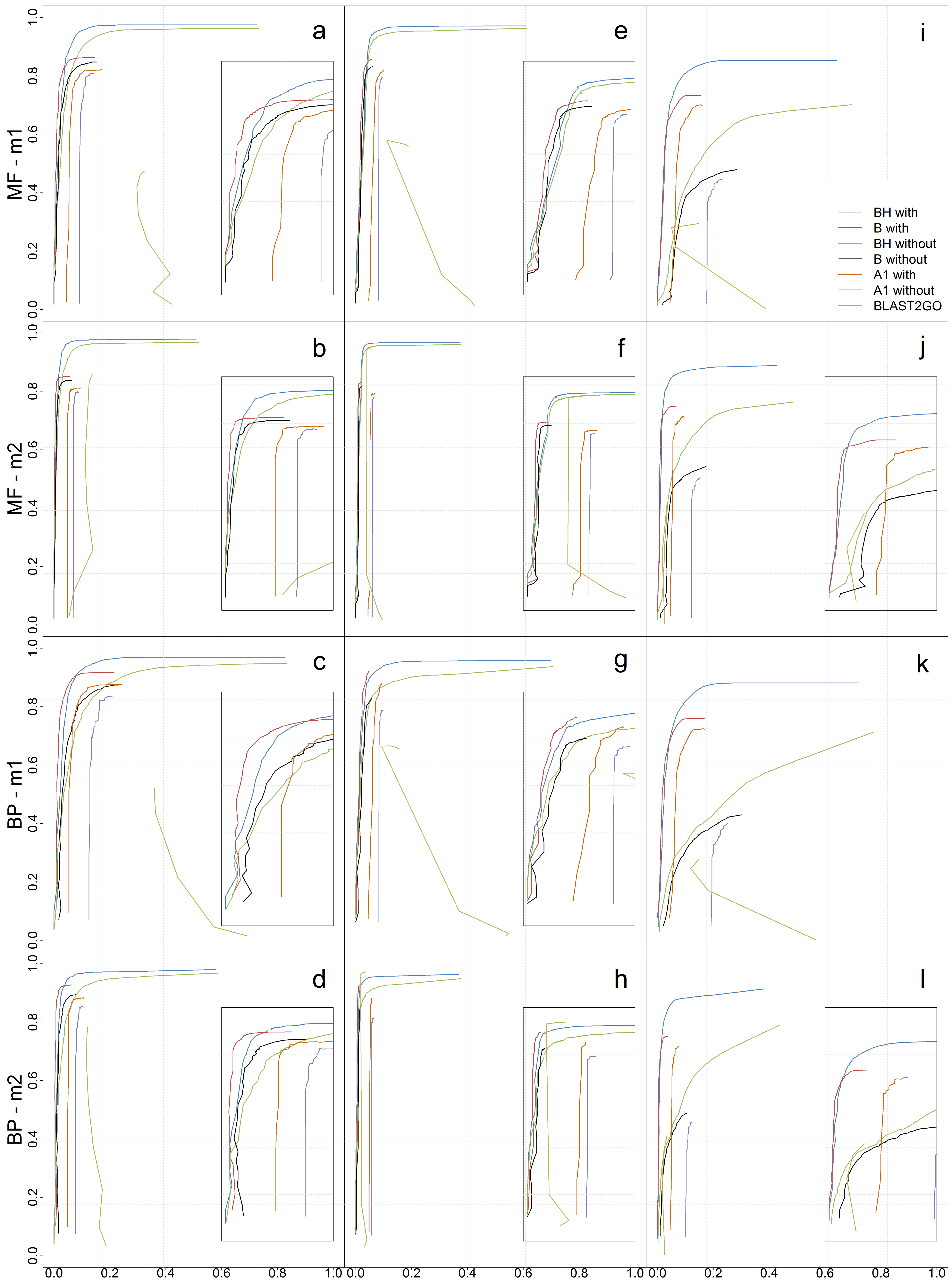

Supplement: Additional file 6 — Precision/Recall curves for the Euk, Pro and Yeast datasets. Precision/recall curves for Molecular Function (MF) and Biological Process (BP) calculated with methods m1 and m2 for Euk, Pro and Yeast test sets. [file 1471-2105-13-S4-S14-S6.pdf]
