# Supplementary material for: Argot2: a large scale function prediction tool relying on semantic similarity of weighted Gene Ontology terms
Source: BMC Bioinformatics. 2012 Mar 28;13(Suppl 4):S14. doi: 10.1186/1471-2105-13-S4-S14 (PMC3314586; doi:10.1186/1471-2105-13-S4-S14)
Supplement: Additional file 7 — CAFA guidelines explanation. Document that explains m1 and m2 methods using a simple example. [file 1471-2105-13-S4-S14-S7.pdf]

CAFA guidelines explanation

1. Data for the example

Let P1 be a protein and GO:0000001, …, GO:0000007 be seven GO terms whose relationships are depicted in Figure 1, together with their root GO:0008150. Terms GO:0000001 and GO:0000005 (in blue) are the correct annotations for P1, while terms GO:0000004, GO:0000005 and GO:0000006 (circled in red) are those retrieved by Argot2.

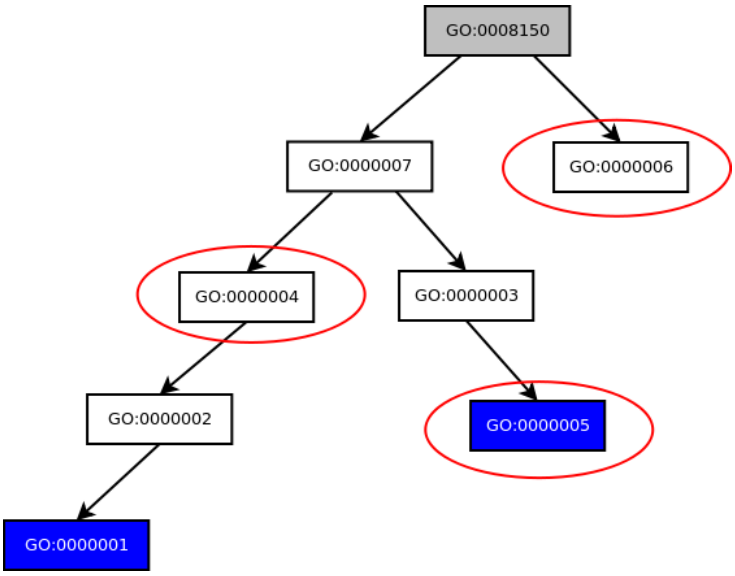

Figure 1: the GO terms for the example and their relationships. The terms circled in red are those retrieved by the tested algorithm; in blue the correct terms.

2. The methods m1 and m2

Method m1 (without propagation)

Method m1 considers true positives (TP) only the exact matches, i.e. the blue terms in Figure 1. According to the previous data, true positives, false positives (FP) and false negatives (FN) are:

	GO IDs	Total
**TP**	5	1
**FP**	4,6	2
**FN**	1	1

For P1 we can compute Precision *P* and Recall *R* as P = TP / (TP + FP) = 0.333 and R = TP / (TP + FN) = 0.5.

Method m2 (with propagation)

Method m2 considers true positives (TP) the exact matches and also all the terms belonging to the intersection of the paths starting from correct and from retrieved terms up to the root; therefore in this case we also consider as true positives terms GO:0000003, GO:0000007 and GO:0008150 that belong to the paths starting from terms GO:0000001, GO:0000004, GO:0000005 and GO:0000006. True positives, false positives (FP) and false negatives (FN) are now:

	Hits	Total
**TP**	3,4,5,7	4
**FP**	6	1
**FN**	1,2	2

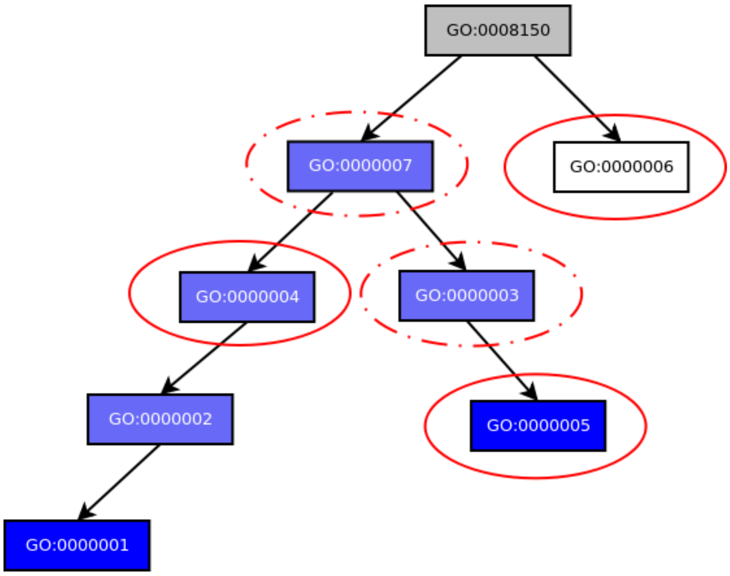

Figure 2: the GO terms for the example after the propagation to the root. In light blue are shown the terms propagated from true annotations; the dashed red circles indicate terms propagated from predicted annotations.

Precision *P* and Recall *R* are now P = TP / (TP + FP) = 0.4 and R = TP / (TP + FN) = 0.667.

3. *Protein-centric evaluation*

The CAFA guidelines proposed a mandatory protein-centric evaluation and an optional annotation-centric evaluation. We followed the first one, that is we averaged Precision and Recall among all proteins.

Example scenario and its evaluation by CAFA criteria

In the following we present a simple scenario to explain how methods m1 and m2 of CAFA guidelines were applied.

Given data

Gene Ontology

Let us build a hypothetical scenario with two proteins (Protein 1 and Protein 2). GO IDs are represented by the generic GO:000008150 plus terms that go from GO:0000001 to GO:0000013 (terms 0, …, 13 in the following). These 14 GO terms have the GO structure shown in Figure 3.

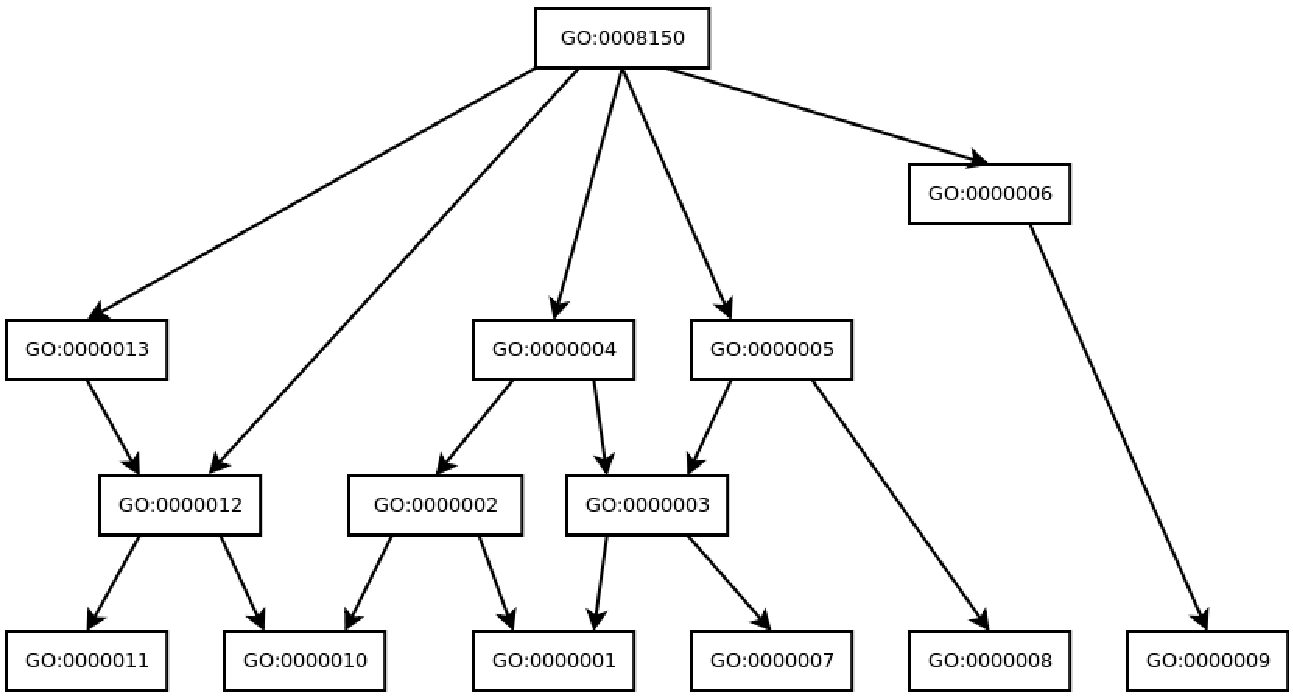

Figure 3: the hypothetical Gene Ontology structure for the examples.

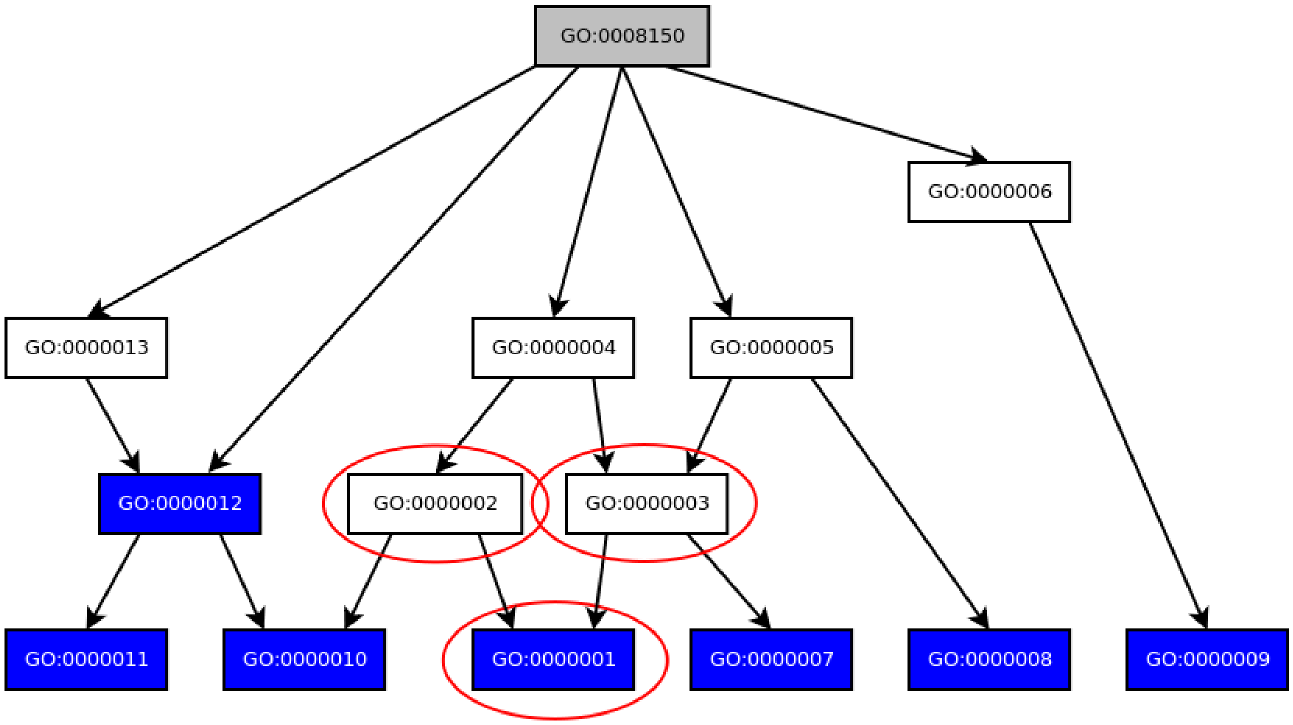

Figure 4: the GO terms for Protein 1 and their relationships (m1). The terms circled in red are those retrieved by the tested algorithm; in blue the correct terms.

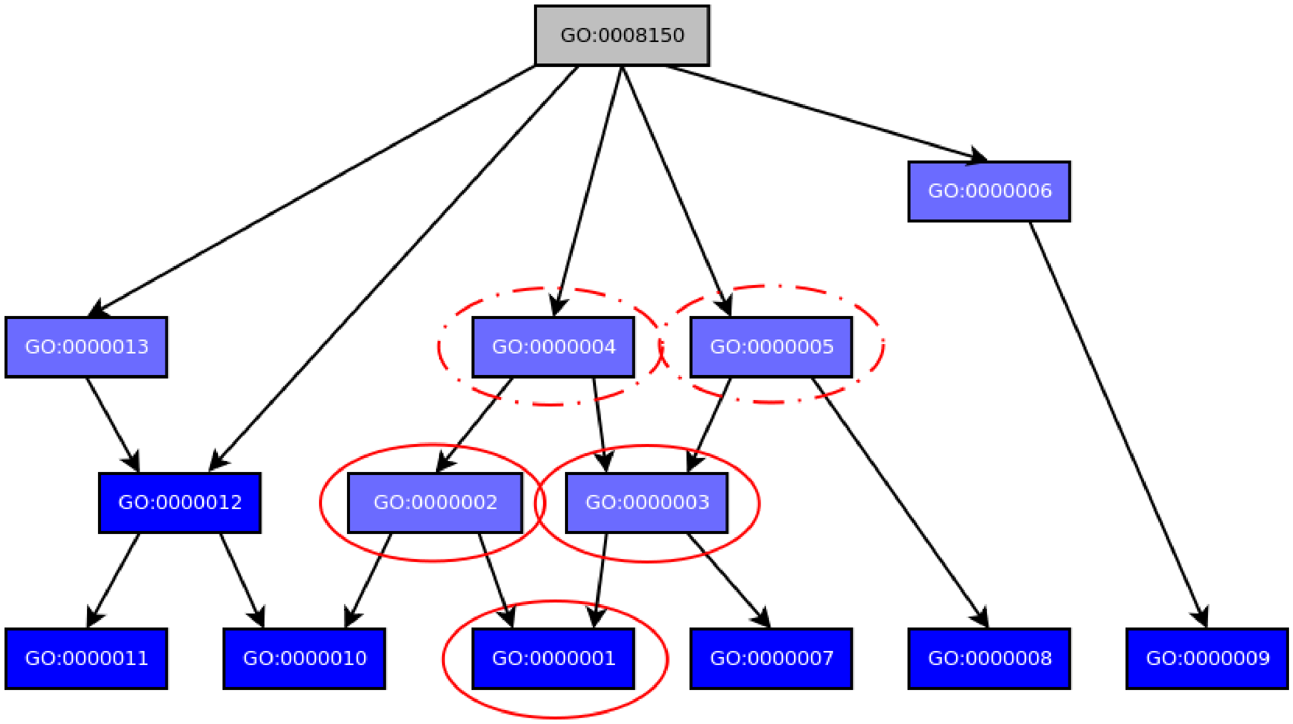

Figure 5: the GO terms for Protein 1 after the propagation to the root (m2). In light blue are shown the terms propagated from true annotations; the dashed red circles indicate terms propagated from predicted annotations.

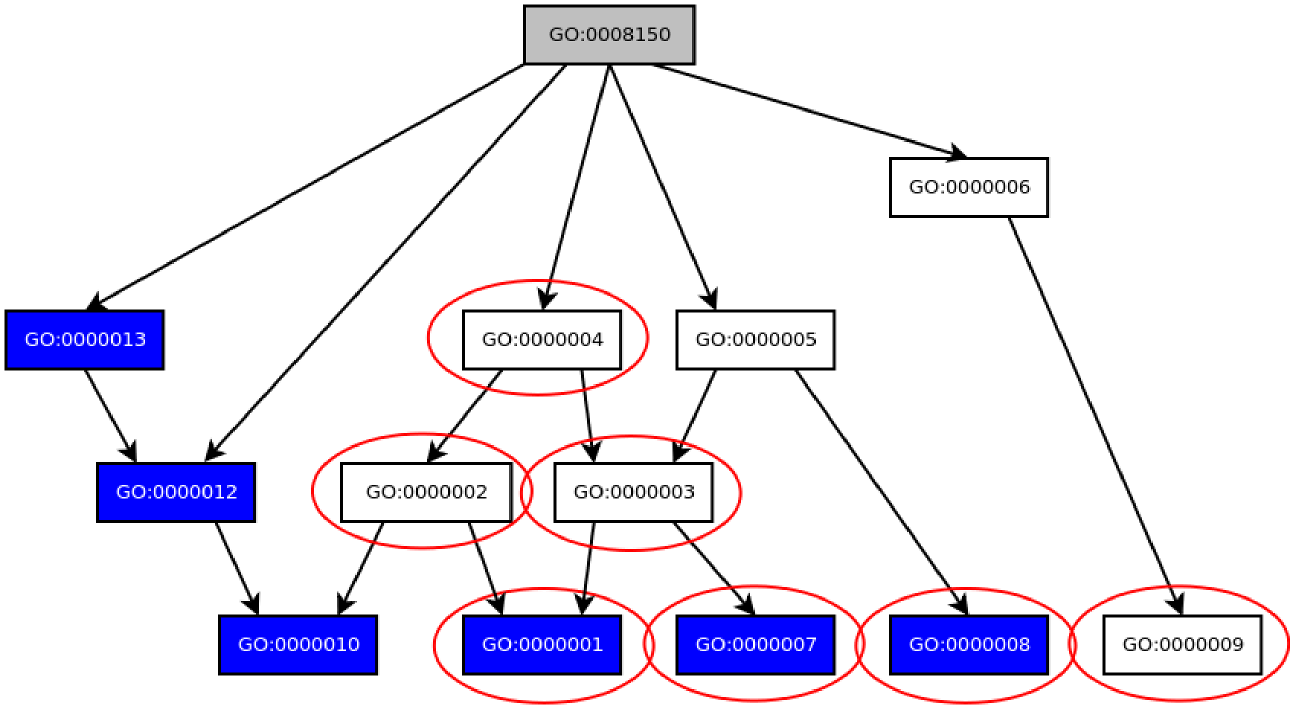

Figure 6: the GO terms for Protein 2 and their relationships (m1). The terms circled in red are those retrieved by the tested algorithm; in blue the correct terms.

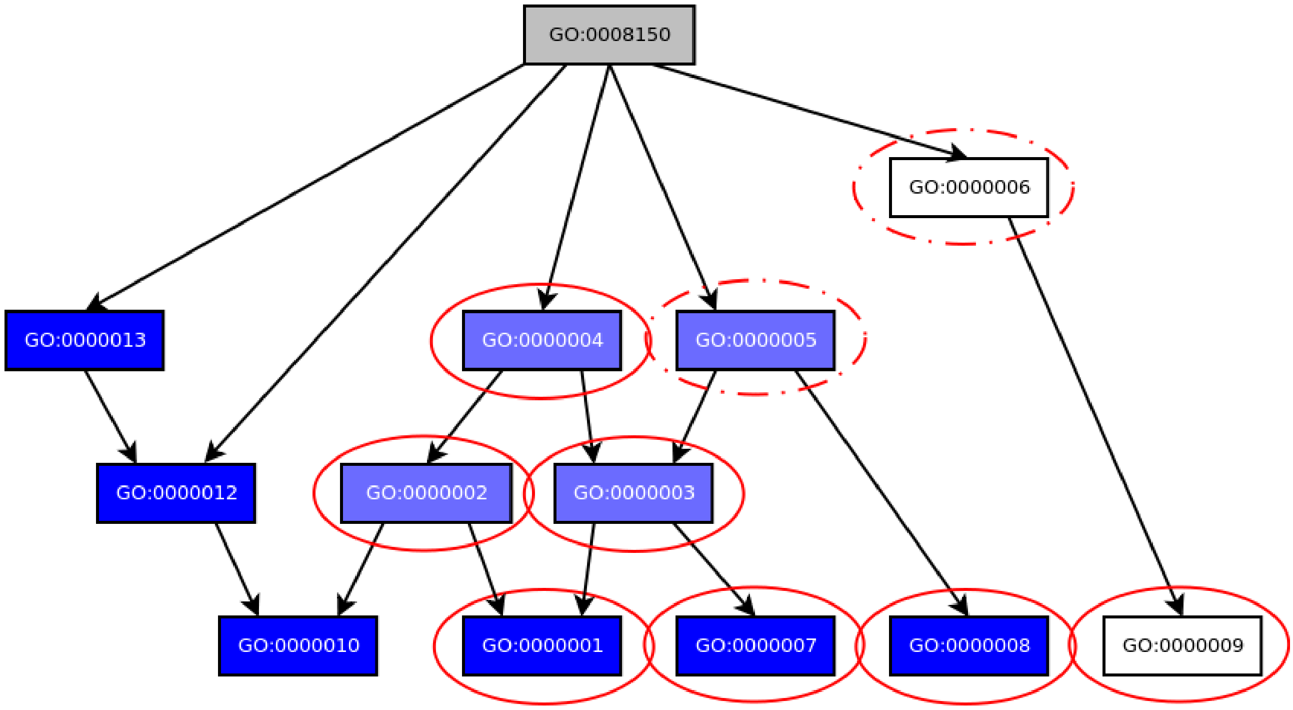

Figure 7: the GO terms for Protein 2 after the propagation to the root (m2). In light blue are shown the terms propagated from true annotations; the dashed red circles indicate terms propagated from predicted annotations.

Evaluation

Schemata in Figures 8-9 and Figures 10-11 represent the scenarios for method m1 and method m2, respectively. Notice that the root term (GO:0008150) is never used in the following considerations. Hereafter, the "Universe of GO terms" is defined as the union of real annotations and the retrieved terms.

Method m1 (without propagation)

Method m1 considers true positives (TP) only the exact matches. According to the previous data, true positives, false positives (FP) and false negatives (FN) for Protein 1 are:

Protein 1	Hits	Total
**TP**	1	1
**FP**	2,3	2
**FN**	7,8,9,10,11,12	6

while results for Protein 2 are:

Protein 2	Hits	Total
**TP**	1,7,8	3
**FP**	2,3,4,9	4
**FN**	10,12,13	3

Starting from these results it is easy to compute precision and recall, by applying their definitions (reported in the paper), and to average these measures between Protein 1 and Protein 2. The final results are $Precision_{m1} = 0.381$, $Recall_{m1} = 0.321$.

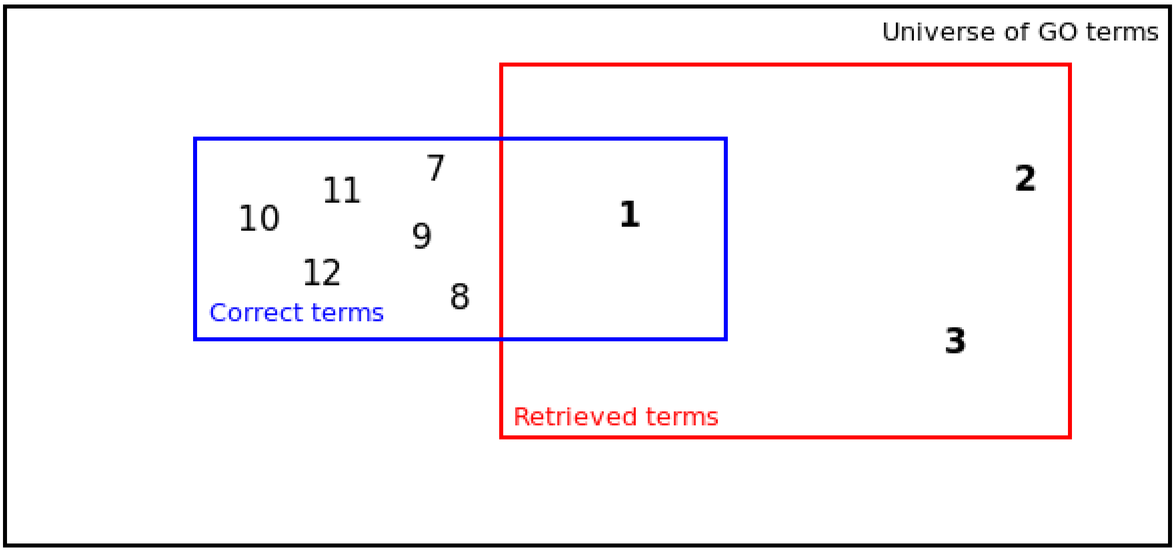

Figure 8: the GO terms scenario for Protein 1 and m1.

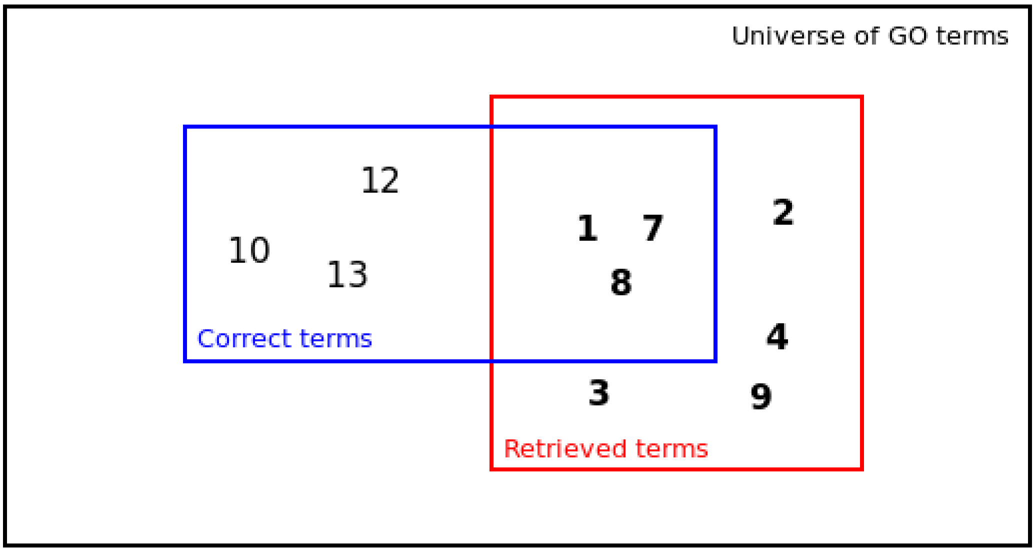

Figure 9: the GO terms scenario for Protein 2 and m1.

Method m2 (with propagation)

Method m2 considers the exact matches and also all the terms belonging to the path from the term itself to the root. The results for Protein 1 are:

	Hits	Total
**TP**	1,2,3,4,5	5
**FP**	-	0
**FN**	6,7,8,9,10,11,12,13	8

while results for Protein 2 are:

	Hits	Total
**TP**	1,2,3,4,5,7,8	7
**FP**	6,9	2
**FN**	10,12,13	3

By applying the definitions of precision and recall and by averaging these measures between Protein 1 and Protein 2 we obtain $Precision_{m2}$ = 0.889, $Recall_{m2}$ = 0.543.

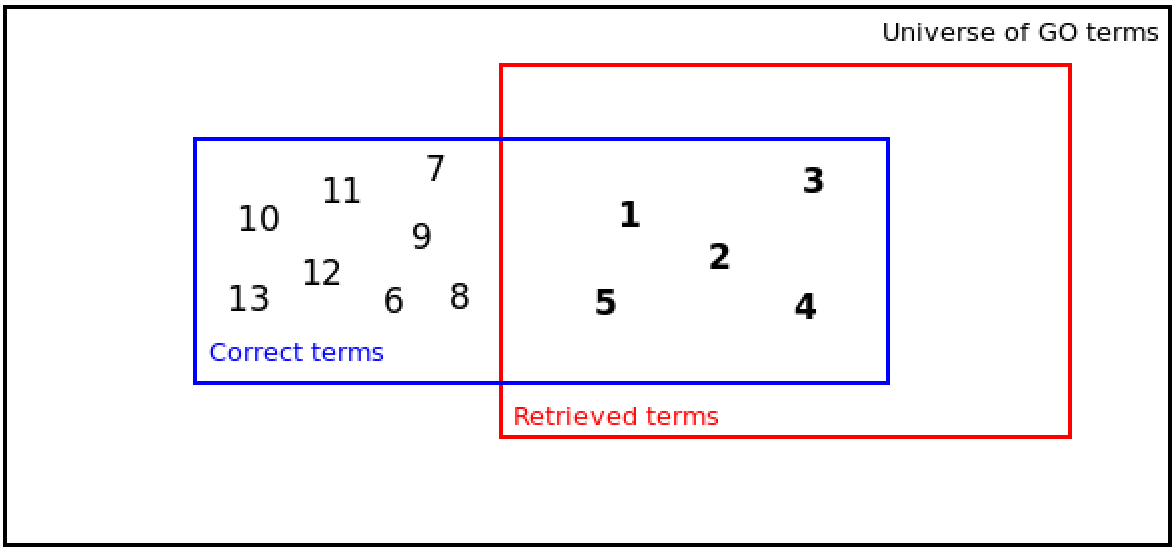

Figure 10: the GO terms scenario for Protein 1 and m2.

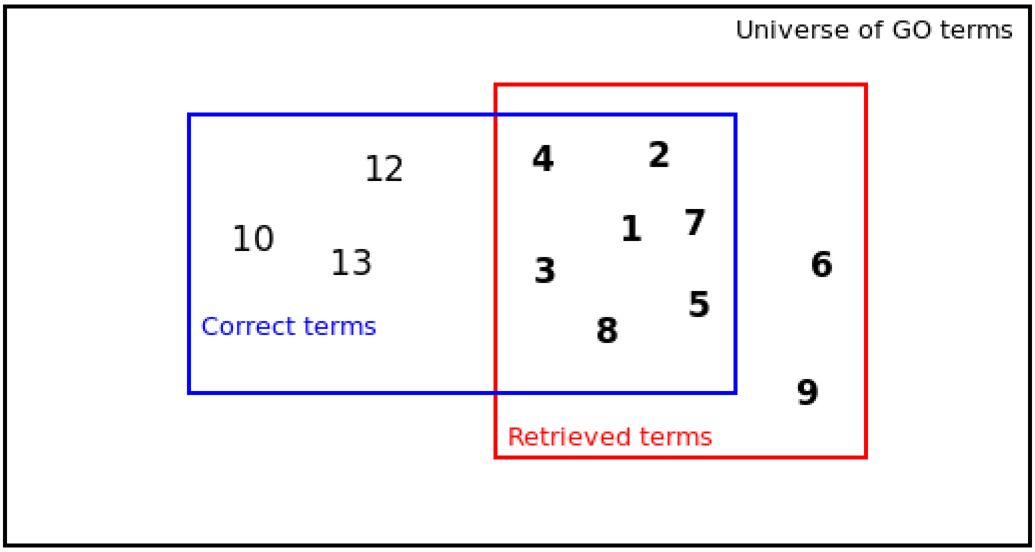

Figure 11: the GO terms scenario for Protein 2 and m2.
